# The RUNX1b Isoform Defines Hemogenic Competency in Developing Human Endothelial Cells

**DOI:** 10.3389/fcell.2021.812639

**Published:** 2021-12-16

**Authors:** Sara Menegatti, Bethany Potts, Eva Garcia-Alegria, Roberto Paredes, Michael Lie-A-Ling, Georges Lacaud, Valerie Kouskoff

**Affiliations:** ^1^ Developmental Hematopoiesis Group, Faculty of Biology, Medicine and Health, The University of Manchester, Manchester, United Kingdom; ^2^ CytoSeek Ltd., Bristol, United Kingdom; ^3^ Stem Cell Process Development, Adaptimmune Ltd., Abingdon, United Kingdom; ^4^ Cancer Research UK Stem Cell Biology Group, Cancer Research UK Manchester Institute, The University of Manchester, Macclesfield, United Kingdom

**Keywords:** hESC = human embryonic stem cell, hematopoiesis, hemogenic endothelial, Runx1, blood

## Abstract

The transcription factor RUNX1 is a master regulator of blood cell specification. During embryogenesis, hematopoietic progenitors are initially generated from hemogenic endothelium through an endothelium-to-hematopoietic transition controlled by RUNX1. Several studies have dissected the expression pattern and role of RUNX1 isoforms at the onset of mouse hematopoiesis, however the precise pattern of RUNX1 isoform expression and biological output of RUNX1-expressing cells at the onset of human hematopoiesis is still not fully understood. Here, we investigated these questions using a RUNX1b:VENUS RUNX1c:TOMATO human embryonic stem cell line which allows multi-parameter single cell resolution *via* flow cytometry and isolation of RUNX1b-expressing cells for further analysis. Our data reveal the sequential expression of the two RUNX1 isoforms with RUNX1b expressed first in a subset of endothelial cells and during the endothelial to hematopoietic transition while RUNX1c only becomes expressed in fully specified blood cells. Furthermore, our data show that RUNX1b marks endothelial cells endowed with hemogenic potential and that RUNX1b expression level determines hemogenic competency in a dose-dependent manner. Together our data reveal the dynamic of RUNX1 isoforms expression at the onset of human blood specification and establish RUNX1b isoform as the earliest known marker for hemogenic competency.

## Introduction

In all species studied to date, the hematopoietic system is established during embryonic life in successive waves, each characterized by their timing of emergence, anatomical location and type of progenitors generated (Lacaud and Kouskoff, 2017; Dzierzak and Bigas, 2018). Using a variety of experimental approaches, it was shown that in vertebrate species all blood progenitors initially emerge from endothelial cells with hemogenic properties (Jaffredo et al., 1998; Zovein et al., 2008; Boisset et al., 2010). This endothelial cell population termed hemogenic endothelium (HE) gives rise to blood cells through an endothelial to hematopoietic transition (EHT) during which the endothelial program is shutdown while the hematopoietic program is activated (Gritz and Hirschi, 2016). While it is well established that some endothelial cells possess a hemogenic potential, this hemogenic competency is only defined retrospectively in assays in which the endothelial population is further cultured to reveal or not its hematopoietic potential. No specific marker to date, with the potential exception of CD73, has allowed to define hemogenic competency within the endothelial cell population at the onset of human blood development. However, CD73 was used as a negative marker with CD73^neg^ endothelial cells enriched in HE potential relative to CD73^pos^ cells that represent fully committed endothelial cells (Choi et al., 2012; Ditadi et al., 2015). Choi and colleagues defined CD73^neg^ cells as precursors of both hematopoietic and endothelial cells with day 5 Ve-cad^pos^CD73^neg^ cells able to form vascular tubes in Matrigel matrix. Therefore, although narrowing down the differentiation potential, this negative selection is not sufficient to distinguish solely hemogenic endothelium.

During embryonic development, the transcription factor RUNX1 was shown to be critical for blood cell emergence in murine (Chen et al., 2009; Lancrin et al., 2009) and human (Bruveris et al., 2020) model systems. The expression and requirement of the main RUNX1 isoforms, RUNX1b and RUNX1c, was characterized in murine systems with RUNX1b expressed in HE and critical for establishing the hematopoietic system while RUNX1c expression was only upregulated in committed blood progenitors (Sroczynska et al., 2009; Bee et al., 2010). The expression and requirement of RUNX1 isoforms during human hematopoietic development have also been analyzed in a few studies to date. The expression of RUNX1 isoforms was first analyzed by PCR in differentiated human embryonic stem cells (hESCs); RUNX1b was found expressed throughout differentiation, while RUNX1c was upregulated later, alongside CD34, CD43 and TAL1 by day 12–16 of differentiation (Challen and Goodell, 2010). A refined analysis of RUNX1c expression was performed using a hESC RUNX1c-GFP knock-in line and revealed that at day 8 of differentiation RUNX1c became expressed during EHT but not in the endothelial population prior to this transition (Ditadi et al., 2015; Ng et al., 2016). A study using a transgenic RUNX1c-Tomato hESC line reported Tomato expression in HE (Angelos et al., 2018). As this study used a transgenic construction carrying the P1 promoter and the +24 enhancer, it was not fully clear which RUNX1 isoform expression was monitored. Finally, a study by Navarro-Montero and colleagues followed *RUNX1b* and *RUNX1c* expression by PCR over time in differentiating human EBs, showing the differential expression of these two isoforms during blood cell emergence and maturation (Navarro-Montero et al., 2017).

In the present study, we followed simultaneously the expression of RUNX1b and RUNX1c isoforms using hESCs genetically modified to express fluorescence reporters inserted in front of the P2 and P1 promoters. This novel hESC line allows multi-parameter single cell resolution *via* flow cytometry and isolation of RUNX1b-expressing cells for further analysis. While this type of study has been performed using mouse models previously, it remains essential to assess the differences and similarities of this developmental process in mouse and human as any regenerative medicine benefits from ESCs in the field of hematology will involve the use of human ESCs. Therefore, it is critical to fully understand the regulation of blood cell emergence in the context of human embryonic development. Here, we describe the sequential expression of the two RUNX1 isoforms during blood cell emergence. RUNX1b was first expressed in endothelial cells, this expression was maintained in maturing blood cells. In contrast, RUNX1c only became expressed in fully committed blood cells. These data resolve any possible controversy on RUNX1 isoform expression during human hematopoietic differentiation as the expression of both isoforms was assessed concurrently in the same cells over the course of the differentiation process. Furthermore, we demonstrate functionally that RUNX1b-expressing endothelial cells were highly enriched in HE and that hemogenic competency increased with RUNX1b expression level. Together our data reveal the dynamic of RUNX1 isoform expression at the onset of human blood specification and establishes that, as in mouse, RUNX1b isoform is the earliest known marker for hemogenic competency.

## Materials and Methods

### Generation of RUNX1 Reporter hESC Lines

Full details of the RUNX1 reporter generation are available online. Briefly, VENUS and TOMATO reporter cDNA were successively introduced in the RUNX1 locus using the CRISPR/cas9 approach with specific guide RNA and template constructs containing 5′ and 3′ homology arms surrounding the genomic region to be modified (Supplementary Figure S1). After transduction, clones were selected with G418, screened by PCR and the neomycin resistance gene was removed *via* FLP-FRT excision in the positive clones. Individual ESC clones were tested for heterozygous insertion of the reporter constructs by PCR for both endogenous and modified locus. Additionally, individual ESC clones were tested to determine if the two reporter constructs were inserted in cis or in trans on the RUNX1 locus, this was performed by RT-PCR to detect the transcription of both RUNX1c and RUNX1b isoforms on mRNA extracted from differentiated ESCs.

### ESCs Maintenance and Differentiation

Human ESCs (Man5 line (Garcia-Alegria et al., 2018)) were thawed and maintained on mitotically inactivated MEFs in Knock-Out DMEM media (Thermo Fisher Scientific) supplemented with 20% Knock-Out Serum Replacement (Thermo Fisher Scientific), 1% Minimum Essential Medium (MEM) Non-Essential Amino acid (Thermo Fisher Scientific), 2 mM L-Gln, 25U/ml Pen/Strep, 0.1 mM 2-mercaptoethanol and 8 ng/ml human recombinant bFGF (PeproTech). Before differentiation, hESCs were feeder-depleted by culturing on Geltrex (Thermo Fisher Scientific) for 5 days in TeSR-E8 media (STEMCELL Technologies), supplemented with 25U/ml Pen/Strep. To generate EBs, hESCs were treated with EDTA and gently dissociated with EZPassage Tool (Thermo Fisher Scientific). Cell clumps were resuspended in StemPro-34 (Gibco) supplemented with 2 mM L-Gln, 50U/ml Pen/Strep, 150 ug/ml Transferrin, 50 ug/ml Ascorbic Acid, 4.5 × 10^–4^ M MTG, Geltrex (1:200), 10uM ROCK inhibitor, 10 ng/ml BMP4, plated in low-attachment dishes and incubated at 37°C 5% O_2_ 5% CO_2_. After 24 h, 5 ng/ml bFGF was added to each dish. At day 2, EBs were collected and resuspended in stemPro-34 supplemented with 2 mM L-Gln, 50U/ml Pen/Strep, 150ug/ml Transferrin, 50ug/ml Ascorbic Acid, 4.5 × 10^–4^ M MTG, 10 ng/ml BMP4, 5 ng/ml bFGF, 0.9 ng/ml Activin A. At day 4, EBs were collected and resuspended in stemPro-34 supplemented with 2 mM L-Gln, 50U/ml Pen/Strep, 150ug/ml Transferrin, 50ug/ml Ascorbic Acid, 4.5 × 10^–4^ M MTG, 5 ng/ml bFGF, 12 ng/ml VEGF in StemPro-34). Further details are available in Star Protocol (Garcia-Alegria et al., 2021).

### Hemogenic Endothelium Sort and Culture

After 6 days of culture, EBs were collected, disaggregated and stained with APC-eF780 conjugated anti-human CD31 (Thermo Fisher Scientific), PE-conjugated anti-human CD144 (BioLegend), PerCP-eF710-conjugated anti-human CD43 (Thermo Fisher Scientific) and Hoechst 33258 (Thermo Fisher Scientific). Live CD31^+^CD144^+^CD43^−^ cells were sorted and replated on gelatin-coated dishes in StemSpan (STEMCELL Technologies) supplemented with 5 ng/ml VEGF, 5ng bFGF, 25 ng/ml IGF1, 25 ng/ml IGF2, 50 ng/ml SCF, 50 ng/ml TPO, 5 ng/ml IL-11, 20 ng/ml Flt3-L (all cytokines from PeproTech). Additional materials and methods are available in Supplemental Text.

## Results

### RUNX1b and RUNX1c Isoforms Mark Different Stages of Blood Cell Emergence in Humans

**FIGURE 1:**
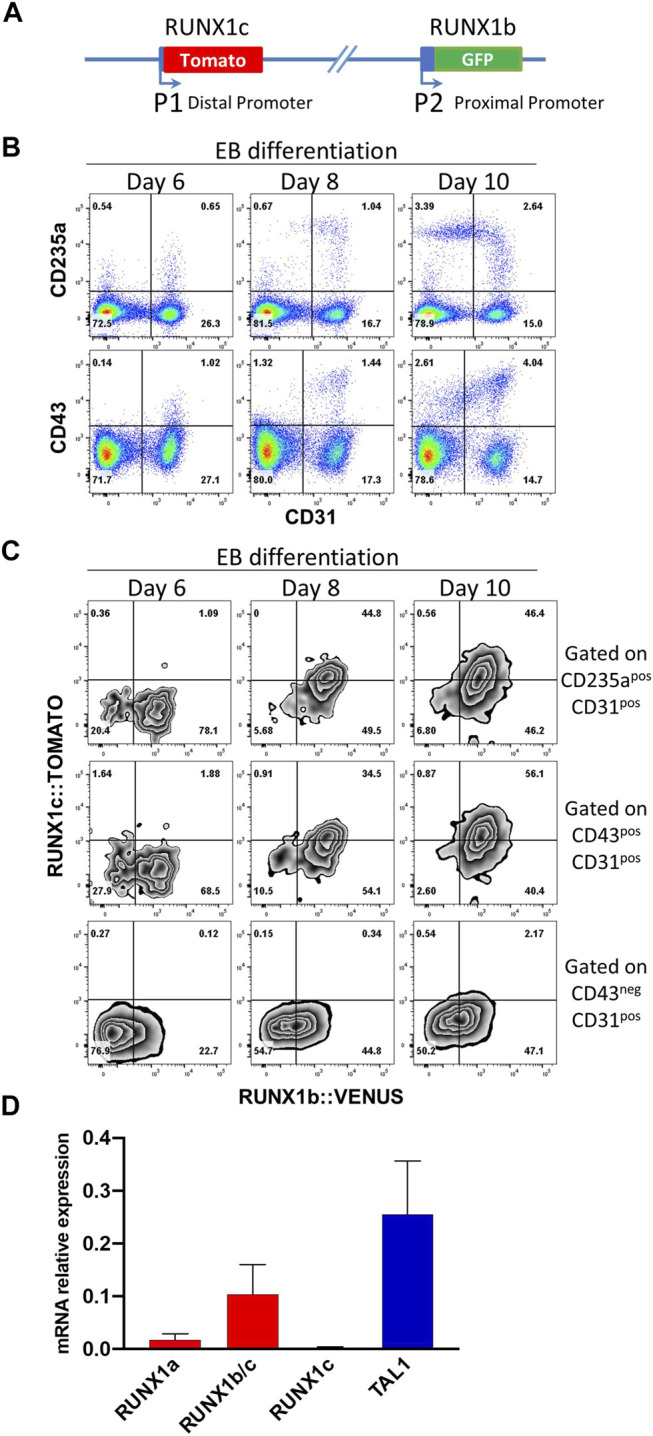
RUNX1b and RUNX1c mark different stages of blood specification. **(A)** Schematic representation of the modified *RUNX1* locus in human ESCs. **(B)** Flow cytometry analysis of embryoid body (EB) cells at day 6, 8 and 10 of differentiation for the indicated cell surface markers. **(C)** Flow cytometry analysis for RUNX1b:VENUS and RUNX1c:TOMATO in CD31^+^CD43^−^ endothelial cells, CD31^+^CD43^+^ and CD31^+^CD235a^+^ hematopoietic cells. **(D)** Expression of the indicated genes by qRT-PCR in CD31^+^CD43^−^ cell populations sorted from day 6 EBs. Data are presented as mean +/− SD of 3 independent experiments. All data are representative of at least 3 independent experiments. Similar expression pattern for RUNX1b:VENUS and RUNX1c:TOMATO were observed in two independent hESC clones.

To determine the dynamic of RUNX1 main isoform expression at the onset of blood cell development, we generated hESC clones carrying a H2B-VENUS reporter under the control of the proximal P2 promoter which controls *RUNX1b* transcription and a TOMATO reporter under the control of the distal P1 promoter which controls *RUNX1c* transcription, with both reporters inserted in cis on the same RUNX1 allele (Figure 1A, Supplementary Figure S1). This double reporter hESC line, allowing to follow simultaneously the expression of both isoforms, was induced to differentiate *in vitro via* embryoid body (EB) formation to generate blood cells as previously described (Garcia-Alegria et al., 2018). The expression of RUNX1b:VENUS and RUNX1c:TOMATO was monitored in EBs by flow cytometry during the course of the differentiation process. By day 6 of differentiation, a CD31^+^ endothelial population was clearly visible with very few cells co-expressing either the hematopoietic markers CD43 or CD235a (Figure 1B), which are the first markers to be upregulated upon hematopoietic specification (Vodyanik et al., 2006). By day 8 of differentiation, the frequencies of CD31^+^ cells committed to hematopoiesis, expressing CD43 and/or CD235a, increased and by day 10, more committed CD235a^+^ and CD43^+^ cells were seen to switch off their CD31 expression. During this differentiation process, we analyzed the expression of the two RUNX1 isoforms in CD31^+^CD43^−^ endothelial cells and in CD31^+^CD43^+^ or CD31^+^CD235a^+^ hematopoietic cells.

While RUNX1b:VENUS expression was readily detected in the endothelial population at day 6 and at later time points, RUNX1c:TOMATO remained undetectable in this population (Figure 1C). In contrast, the expression of both RUNX1 isoforms became detectable over time in the CD31^+^CD43^+^ and CD31^+^CD235a^+^ hematopoietic-committed populations. Interestingly, at day 6, emerging hematopoietic cells only expressed RUNX1b:VENUS while RUNX1c:TOMATO expression, undetectable at day 6, increased progressively over time in the CD43^+^ and CD235a^+^ populations. To independently validate this observation, quantitative RT-PCR was performed for *RUNX1* isoform expression in CD31^+^CD43^−^ cells isolated from day 6 EBs. The use of differential detection for *RUNX1a*, *RUNX1c* and *RUNX1b/c* demonstrated that *RUNX1b* was the predominant mRNA isoform detected in the endothelial population prior to hematopoietic commitment, along with *TAL1* expression as an early marker of cardiovascular development (Figure 1D). Only low levels of the *RUNX1a* isoform were detected at this stage. This is not surprising as, similar to the *RUNX1b* isoform, *RUNX1a* is transcribed from the P2 promoter. Although our GFP reporter cannot distinguish between the expression of these two RUNX1 isoforms, these results suggest that GFP levels at this stage of the differentiation are mainly representative of the RUNX1b isoform and only to a lesser extent of the RUNX1a isoform.

Together, these data demonstrate that RUNX1b is the first RUNX1 isoform expressed in endothelial cells before specification to hematopoiesis marked by CD43 and/or CD235a upregulation. The RUNX1c isoform becomes expressed over time in committed hematopoietic cells but clearly lags behind RUNX1b expression. Hence, these two RUNX1 isoforms are differentially expressed at the onset of blood cell emergence and mark different cell subsets and stages in differentiating hESCs.

### RUNX1b Positive and Negative Endothelial Cells Share a Similar Immuno-Phenotype

**FIGURE 2:**
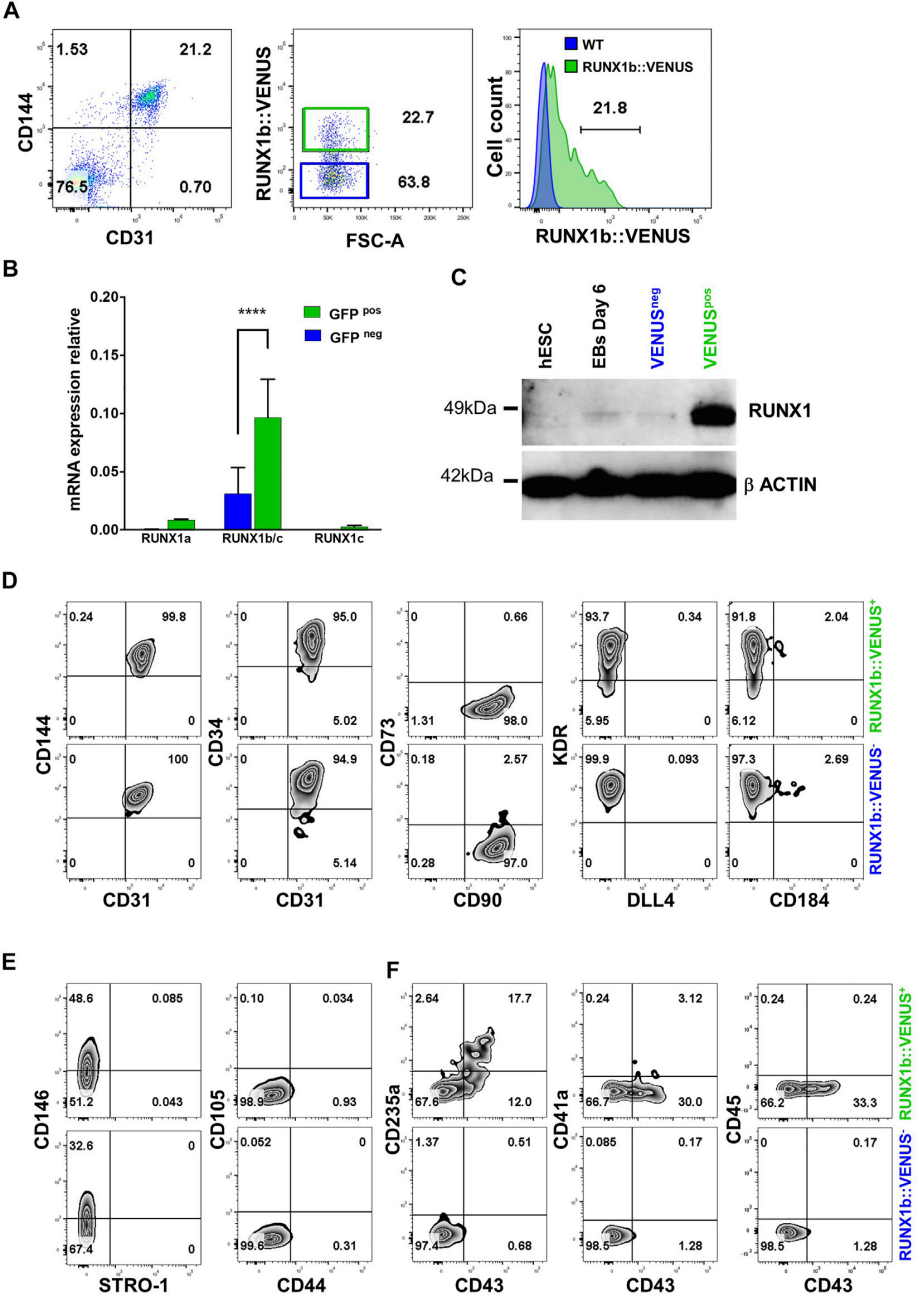
Characterization of the RUNX1b:VENUS population. **(A)** Flow cytometry analysis of CD144 and CD31 expression in day 6 EBs **(left panel)**. RUNX1b:VENUS expression level in the CD144^+^CD31^+^ population **(middle panel)**. RUNX1b:VENUS expression against control auto-fluorescence from CD144^+^CD31^+^ differentiated wild-type hESCs **(right panel)**. **(B)** Expression of the indicated genes by qRT-PCR in VENUS positive and negative CD31 + CD144 + cell populations sorted from day 6 EBs. Data are presented as mean +/− SD of 3 independent experiments. **(C)** Western blot analysis for RUNX1 protein level in the indicated populations. β−ACTIN expression was used as loading control. **(D–F)** Flow cytometry analysis of VENUS positive and negative cell populations at day 6 of differentiation for the indicated cell surface markers. All data are representative of at least 3 independent experiments.

We next focused on the early endothelial population expressing RUNX1b as no characterization or functional assay of this population has been reported to date. First, we isolated RUNX1b:VENUS positive and negative cells within the CD31^+^CD144^+^ population from day 6 EBs to evaluate the reliability and accuracy of the reporter hESC line (Figure 2A). The two cell populations were assessed for *RUNX1b* mRNA expression by qRT-PCR (Figure 2B). This analysis revealed a significant enrichment for the combined *RUNX1b/c* and *RUNX1a* mRNA in the VENUS^+^ fraction, while only background level for *RUNX1c* mRNA was detected, confirming that VENUS expression faithfully enriched for *RUNX1b* transcripts and also for the *RUNX1a* low transcript level. RUNX1 protein levels were assessed by western blotting (Figure 2C), and also revealed a strong enrichment for RUNX1 expression in the VENUS positive cell population.

Next, we compared the two populations expressing or not RUNX1b:VENUS by multi-parameter flow cytometry in day 6 EBs. The expression of commonly used endothelial markers such as CD144, CD34, CD31 and KDR showed little differences between the two populations (Figure 2D). The main notable difference was observed in KDR staining where a fraction of the RUNX1b:VENUS^+^ cells displayed lower KDR expression level. The expression of the endothelial mature marker CD73 was barely detectable in either population, as were the expression of DLL4 and CD184 that have both been previously shown to mark hemogenic endothelial subsets at later stage of differentiation (Ayllón et al., 2015; Ditadi et al., 2015; Uenishi et al., 2018). The expression of CD90 was detected on all CD31^+^CD144^+^ cells, regardless of RUNX1b expression status (Figure 2D). CD90 is expressed by many cell types including mesenchyme, we therefore assessed the expression of several other mesenchyme markers to determine if this population might harbor a wider immuno-phenotype than endothelium. While CD146 was expressed on a subset of cells in both RUNX1b:VENUS^+^ and RUNX1b:VENUS^−^ populations, the expression of CD44, CD105 and STRO-1 was not detected (Figure 2E). In contrast to the endothelial immuno-phenotype, the expression of the early hematopoietic markers CD235a and CD43 was only detected in the RUNX1b:VENUS^+^ population (Figure 2F). At this early stage of differentiation, the expression of the more mature hematopoietic markers, CD41a and CD45, was not observed.

Together, these data demonstrate that the RUNX1b:VENUS hESC reporter line is an accurate tool to track and isolate RUNX1b-expressing cells upon *in vitro* differentiation. Additionally, our immuno-phenotypic characterization of the VENUS positive and negative cells revealed a very homogenous endothelial identity in both populations. Interestingly, only cells within the RUNX1b:VENUS population were shown to express hematopoietic markers, suggesting that RUNX1b expression is a prerequisite for hematopoietic commitment during human development.

### RUNX1b Expression Enriches for Hemogenic Potential

**FIGURE 3:**
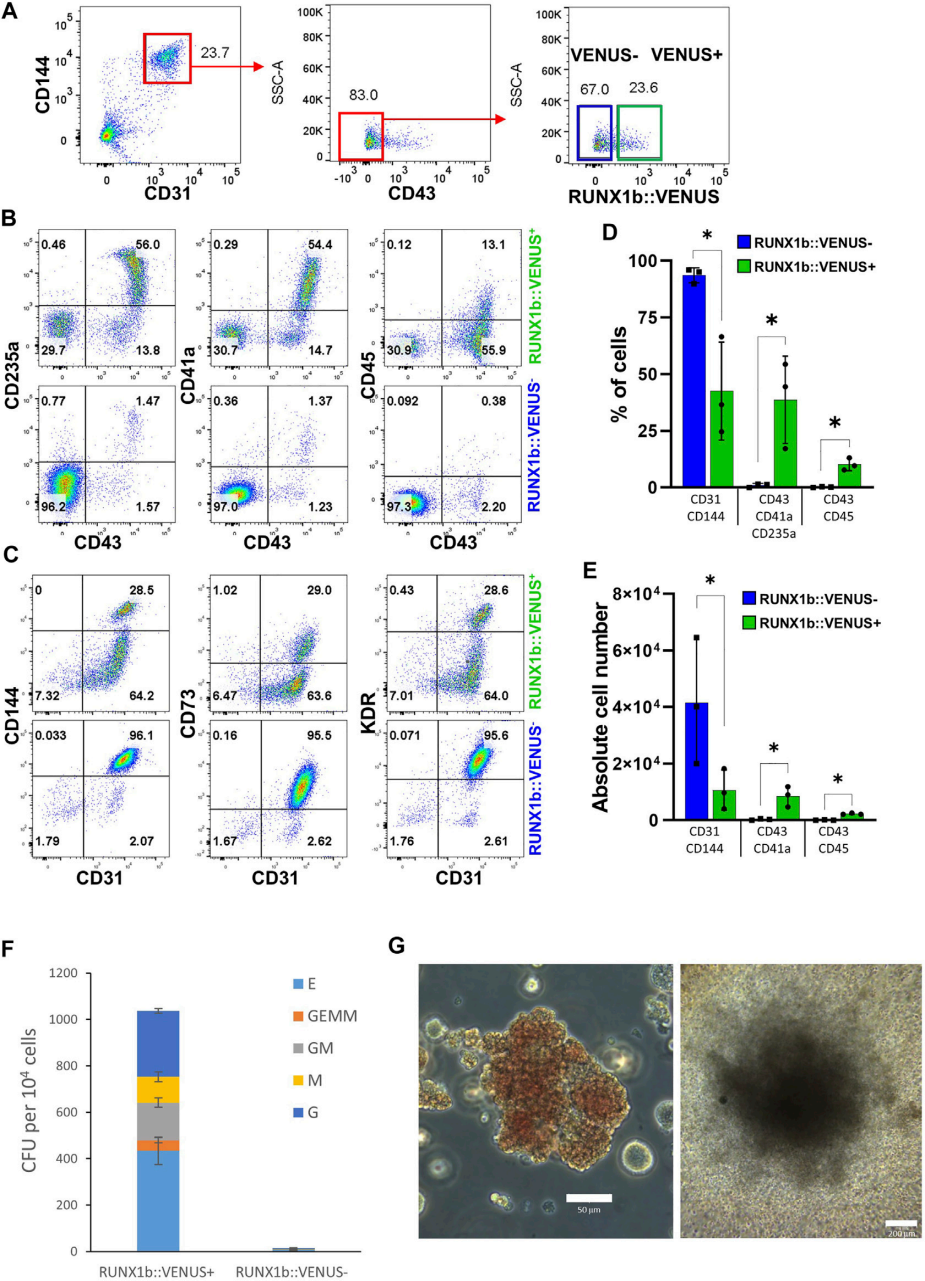
The RUNX1b:VENUS marks hemogenic endothelium. **(A)** Gating strategy used to sort VENUS positive and negative cell populations from day 6 EBs for hemogenic potential analysis. **(B, C)** Flow cytometry analysis for the indicated cell surface markers at day 4 of hemogenic inducing cultures for the sorted VENUS positive and negative cell populations. **(D)** Frequencies and **(E)** absolute numbers of endothelial (CD31^+^CD144^+^) and hematopoietic (CD43^+^CD41a^+^CD235a^+^ and CD43^+^CD45^+^) cells at day 4 of hemogenic inducing cultures from 3 independent experiments, data are presented as mean +/− SD. Statistically significant differences were analyzed with unpaired *t*-test *p < 0.05. **(F)** Clonogenic potential of sorted VENUS positive and negative cell populations after 4 days of hemogenic inducing cultures. Data are presented as colony forming unit (CFU) for 10^4^ cells as mean +/− SD from 3 independent experiments, each experiment performed in biological triplicates. E: erythroid, GEMM: granulocyte-erythroid-macrophage-megakaryocyte, GM: granulocyte-macrophage, M: macrophage, G: granulocyte. **(G)** Representative photography of Erythroid **(left)** and GM **(right)** colonies, scale bar: 50 and 200 μm, respectively. Cell colony morphology is representative of at least 3 independent experiments.

We previously showed that the endothelial cell population isolated at day 6 of EB differentiation is enriched for hemogenic potential giving rise to both primitive and definitive blood progenitors (Garcia-Alegria et al., 2018). Therefore, we next aimed to determine how this hemogenic potential was distributed across RUNX1b:VENUS positive and negative cells. To this end, the CD144^+^CD31^+^CD43^−^ day 6 EB endothelial population was sorted based on VENUS expression (Figure 3A) and further cultured in hemogenic inducing culture as previously described (Garcia-Alegria et al., 2021). After 4 or 7 days, cells from these cultures were assessed for their expression of hematopoietic and endothelial markers. By day 4, around 50% of cells generated in the RUNX1b:VENUS^+^ culture expressed the hematopoietic markers CD43, CD235a and CD41a while at this stage of differentiation CD45 was only expressed by less than 15% of CD43^+^ hematopoietic cells (Figures 3B,D). In contrast, very few cells (less than 2%) generated in the RUNX1b:VENUS^−^ culture expressed these hematopoietic markers. Indeed, most cells from the RUNX1b:VENUS^−^ culture retained their endothelial identity, and upregulated CD73 expression which indicates further endothelial maturation (Figures 3C,E). Conversely, the frequency of CD73^+^ endothelial cells in the RUNX1b:VENUS^+^ culture was much lower. By day 7 of the culture, the differences were even more striking with hematopoietic cells only persisting and further maturing in the RUNX1b:VENUS^+^ culture (Supplementary Figure S2A). To complement this immuno-phenotypic analysis, cells from day 4 cultures were tested for their capacity to generate hematopoietic colonies in clonogenic assay (Figure 3F). All types of myeloid and erythroid colonies were generated from the RUNX1b:VENUS^+^ cultures while only a few granulocyte colonies were generated from the RUNX1b:VENUS^−^ cultures. Highly proliferative myeloid colonies and large erythroid colonies were readily observed in the RUNX1b:VENUS^+^ derived cultures (Figure 3G). This series of analyses demonstrate that, at day 6 of differentiation, the hemogenic potential is almost exclusively restricted to the endothelial cells expressing the RUNX1b isoform.

### RUNX1b Dose-dependent Control of Hemogenic Competency

**FIGURE 4:**
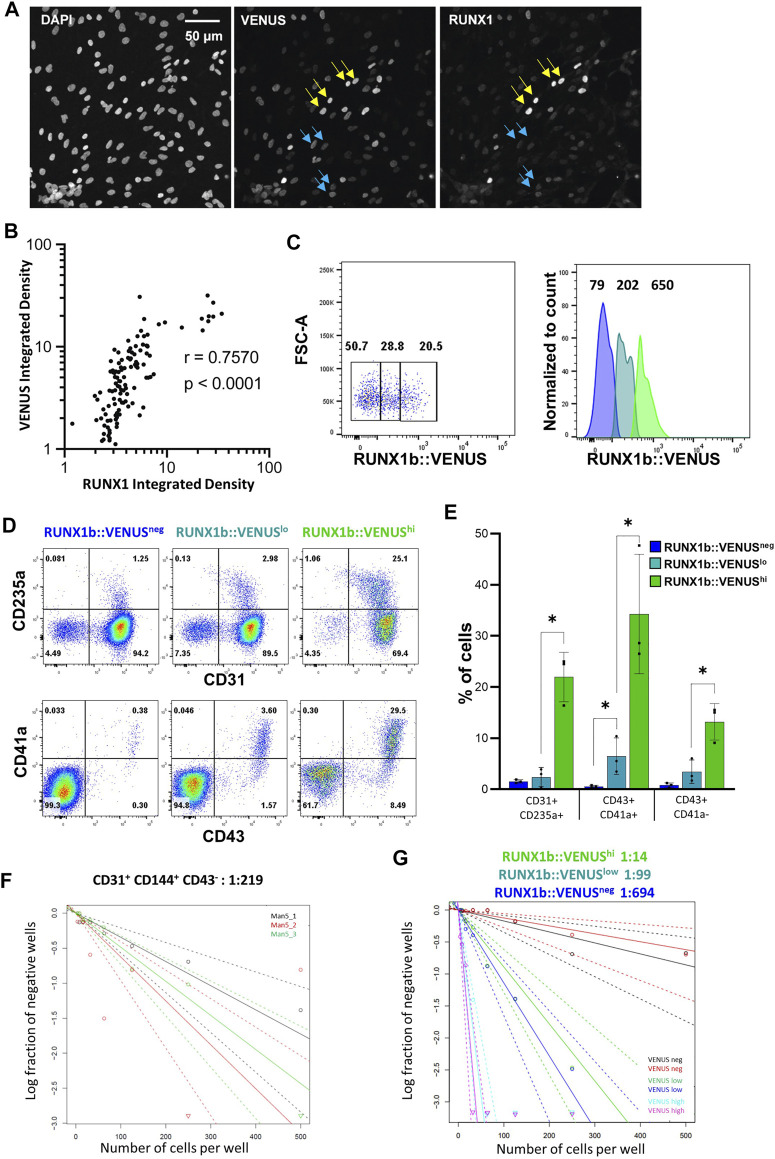
RUNX1b expression level determines hemogenic competency. **(A)** CD31^+^CD43^−^ cells were sorted from day 6 EBs, grown for 1 day on glass slides in hemogenic inducing conditions then stained and analyzed for DAPI, VENUS and RUNX1 expression (yellow arrows: cells with high expression, blue arrows: cells with low expression). VENUS is nuclear as a H2B-VENUS fusion protein reporter construct was used to target the RUNX1b locus. **(B)** For each individual cell, VENUS and RUNX1 signals were integrated, plotted against each other and Pearson correlation coefficient (r) calculated. **(C)** Gating strategy for the isolation of VENUS negative, low and high populations from CD31^+^CD43^−^ day 6 EBs. Numbers on the dot plot represent frequencies (%) and, on the histogram, mean fluorescence intensities. **(D)** Flow cytometry analysis for the indicated cell surface markers at day 4 of hemogenic inducing cultures for the sorted VENUS negative, low and high cell populations. **(E)** Frequencies of hematopoietic cells at day 4 of hemogenic inducing cultures from 3 independent experiments, data are presented as mean +/− SD. Statistically significant differences were analyzed with unpaired *t*-test *p < 0.05. **(F, G)** Limiting dilution analysis of hemogenic potential in the indicated populations. All data are representative of at least 3 independent experiments.

We were intrigued by the expression profile of RUNX1b:VENUS as it forms a continuum of increasing intensity from negative to positive rather than two distinct peaks of positive and negative expression (Figure 2A). First, we assessed to which extent VENUS expression level correlated with RUNX1 endogenous expression level. CD31^+^CD144^+^ cells isolated from day 6 EBs were plated on glass slides, immuno-stained and imaged for DAPI, VENUS and endogenous RUNX1 expression (Figure 4A). For each individual cell, the expression levels of VENUS and RUNX1 were measured, their signal density integrated and plotted against each other (Figure 4B and Supplementary Figure S3). Pearson correlation coefficient was calculated to determine the magnitude of correlation between the two signals. This analysis revealed a very high degree of correlation between VENUS and RUNX1 signal intensity (r = 0.757, *p* < 0.0001), except for the highest values when signal saturation limits the validity of this assay.

Having demonstrated a high degree of correlation between the two signals, we next examined the correlation between RUNX1b:VENUS expression level and hemogenic competency. CD31^+^CD144^+^CD43^−^ cells from day 6 EBs were isolated based on VENUS expression level as negative, low and high cell populations (Figure 4C) and assessed for their hemogenic potential. Equal numbers of cells for each population were replated in hemogenic inducible culture conditions and the outcome of the culture was assessed after 4 and 7 days. Remarkably, the frequency of hematopoietic cells generated in these cultures was in direct correlation with the initial level of RUNX1b:VENUS expression. Very few CD235a^+^, CD43^+^, CD41a^+^ cells were generated in the VENUS^neg^ culture, the frequency of these hematopoietic cells increased in the VENUS^low^ culture and was maximal in the VENUS^high^ culture (Figures 4D,E). The relationship between RUNX1b:VENUS expression level and hemogenic potential became even more pronounced by day 7 of the culture when hematopoietic subsets matured further and increased their CD45 expression (Figure S2B). To determine the number of HE in each endothelial subset, we performed limiting dilution analysis (LDA). The number of HE in CD31^+^CD144^+^CD43^−^ cell populations isolated from wild-type day 6 EBs was 1 in 219 cells (Figure 4F). Similar LDA performed on CD31^+^CD144^+^CD43^−^ cells isolated from RUNX1b:VENUS differentiating hESCs, revealed a striking enrichment for hemogenic potential as RUNX1b level increased from VENUS negative to VENUS high expressing cells. (Figure 4G and Supplementary Figure S4). While VENUS negative cells contained on average 1 HE for 694 cells, this number increased to 1 in 99 in the VENUS low population and 1 in 14 in the VENUS high population.

Together these data demonstrate that increasing RUNX1b level correlates with increasing hemogenic competency and suggests a dose-dependent switch in RUNX1b expression for the acquisition of hemogenic potential by endothelial cells.

## Discussion

Our study investigating the expression of RUNX1 isoforms at the onset of human embryonic hematopoietic emergence has several implications. First, using a double reporter hESC line which allows the simultaneous detection of RUNX1b and RUNX1c, we establish that these two RUNX1 isoforms are sequentially expressed during human blood cell emergence as was previously shown in the mouse system. RUNX1b is expressed first in an endothelial subset prior to hematopoietic specification, and this isoform remains expressed during the transition to blood progenitors as well as in fully committed blood cells. In contrast, RUNX1c is not expressed in endothelial cells or during the transition from endothelium to hematopoiesis as the first blood cells emerging at day 6 of differentiation are still RUNX1c:TOMATO negative. The RUNX1c isoform is progressively upregulated as blood cells mature further. These findings are in line with the observed expression of RUNX1 isoforms during murine blood cell emergence (Sroczynska et al., 2009) and demonstrate the conservation of RUNX1 promoter usage and regulation in mammals during this developmental process. Second, our findings show that RUNX1b marks hemogenic competency in human endothelium much earlier than other previously reported hemogenic markers, such as DLL4 and CD184, which are only detected at a later stage of hESC differentiation (Ayllón et al., 2015; Ditadi et al., 2015; Uenishi et al., 2018). Third, our findings uncover a direct relationship between RUNX1b expression level and hemogenic competency, suggesting that RUNX1b expression level in endothelial cells needs to reach a certain threshold to confer hemogenic potential, shifting the balance from an endothelium to a hematopoietic primed state. In support of this notion, during mouse embryonic development the enforced expression of RUNX1 in newly generated angioblasts was shown to promote hemogenic competency (Eliades et al., 2016; Yzaguirre et al., 2018). Additionally, using knock-out approaches for RUNX1 and its binding partner CBFβ, it was shown in differentiating murine ESCs that RUNX1 dosage was critical for blood formation (Lie-A-Ling et al., 2018). Finally, single-cell RNA-seq analysis of hematopoietic stem cell emergence in murine embryos has implicated RUNX1 dosage as a bottleneck in hemogenic endothelium generation (Zhu et al., 2020). It will be important next to understand how RUNX1b expression is controlled in endothelium, at the transcriptional, translational or post-translational level. Our study describes a unique tool that will help address these questions as it allows enriching for hemogenic potential and investigating the induction and regulation of RUNX1 isoforms. As often is the case with the generation of gene reporters, creating a RUNX1 reporter hESC line resulted in RUNX1 heterozygosis. This may represent issues in certain aspects of RUNX1 regulation or blood cell emergence as it has been previously reported that RUNX1 haploinsufficiency alters the temporal and spatial emergence of hematopoietic stem cells in the developing embryo (Cai et al., 2000; Lacaud et al., 2004). However, *Runx1* heterozygous mice are born at the expected Mendelian ratio and are healthy with no apparent defects in their hematopoietic system, which suggests that overall the hematopoietic system is specified appropriately. In our *in vitro* differentiation system, we observe no differences in the timing of HE emergence and blood cell yield compared to the wt hESCs counterpart.

As all blood progenitors initially derive from endothelial cells with hemogenic properties, understanding what makes an endothelium hemogenic is an area of intense investigation. To gain insight into this seminal question, it will be paramount to define not only how RUNX1b expression is controlled in the hemogenic endothelium itself but also how it is controlled by the microenvironment.

## Data Availability

The original contributions presented in the study are included in the article/Supplementary Material, further inquiries can be directed to the corresponding authors.
