# Supplementary material for: The RUNX1b Isoform Defines Hemogenic Competency in Developing Human Endothelial Cells
Source: Front Cell Dev Biol. 2021 Dec 16;9:812639. doi: 10.3389/fcell.2021.812639 (PMC8716778; doi:10.3389/fcell.2021.812639)
Supplement: Supplementary file 1 [file DataSheet1.pdf]

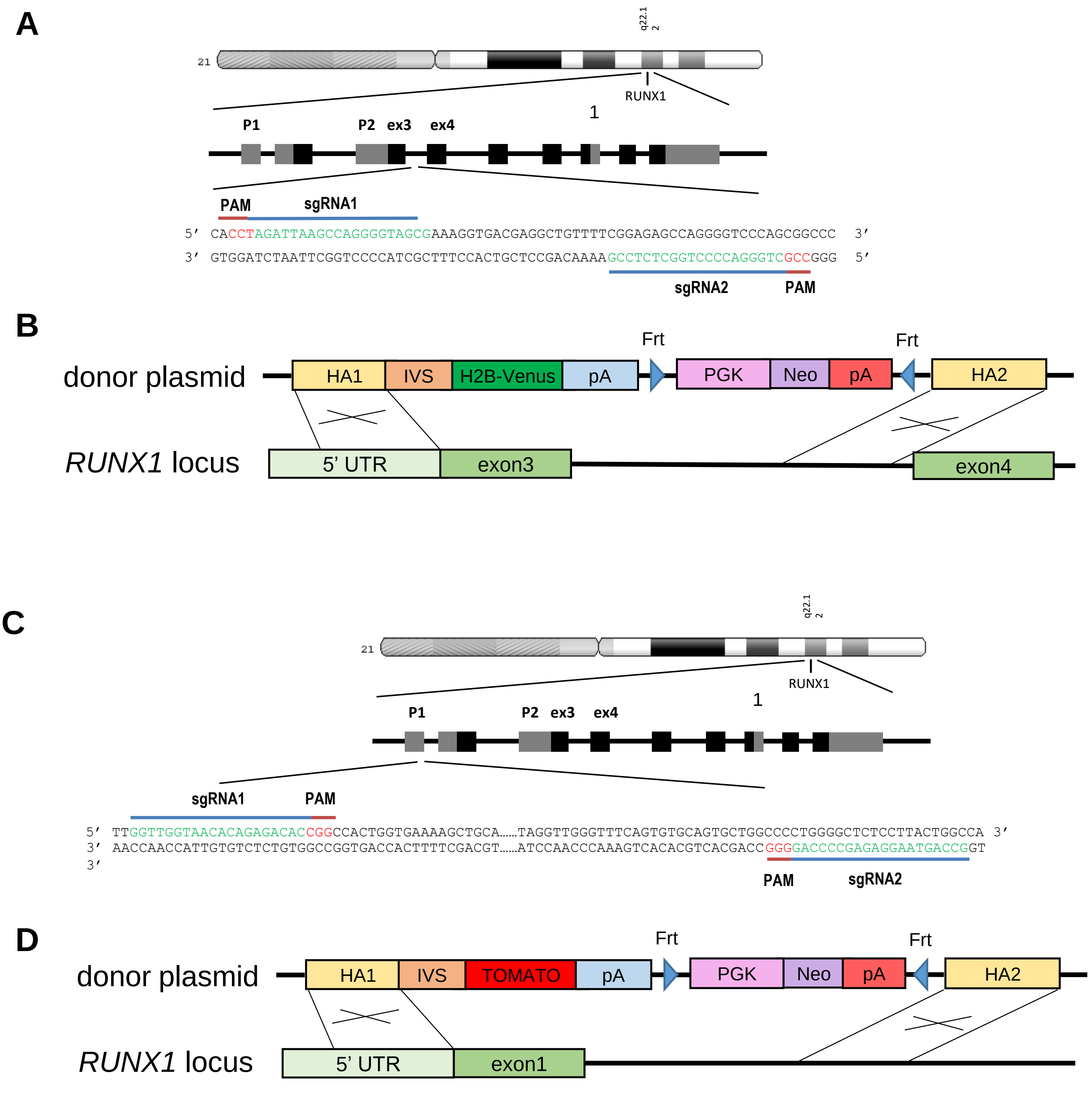

Supplementary Figure S1: **Genetic modification of the RUNX1 locus in hESC**
(A) Representative scheme of CRISPR-Cas9 strategy used to target intron 3 of the *RUNX1* locus. (**B**) Scheme representing the donor plasmid used as a template for homologous recombination to modify the RUNX1b isoform. (**C**) Representative scheme of CRISPR-Cas9 strategy used to target intron 1 of the *RUNX1* locus. (**D**) Scheme representing the donor plasmid used as a template for homologous recombination to modify the RUNX1c isoform. The two homology arms (HA1) for both locus contain identical promoter sequences to the endogenous locus which results in each fluorescent cDNA replacing the respective RUNX1 coding sequence at their ATG codon upon CRISPR modification.

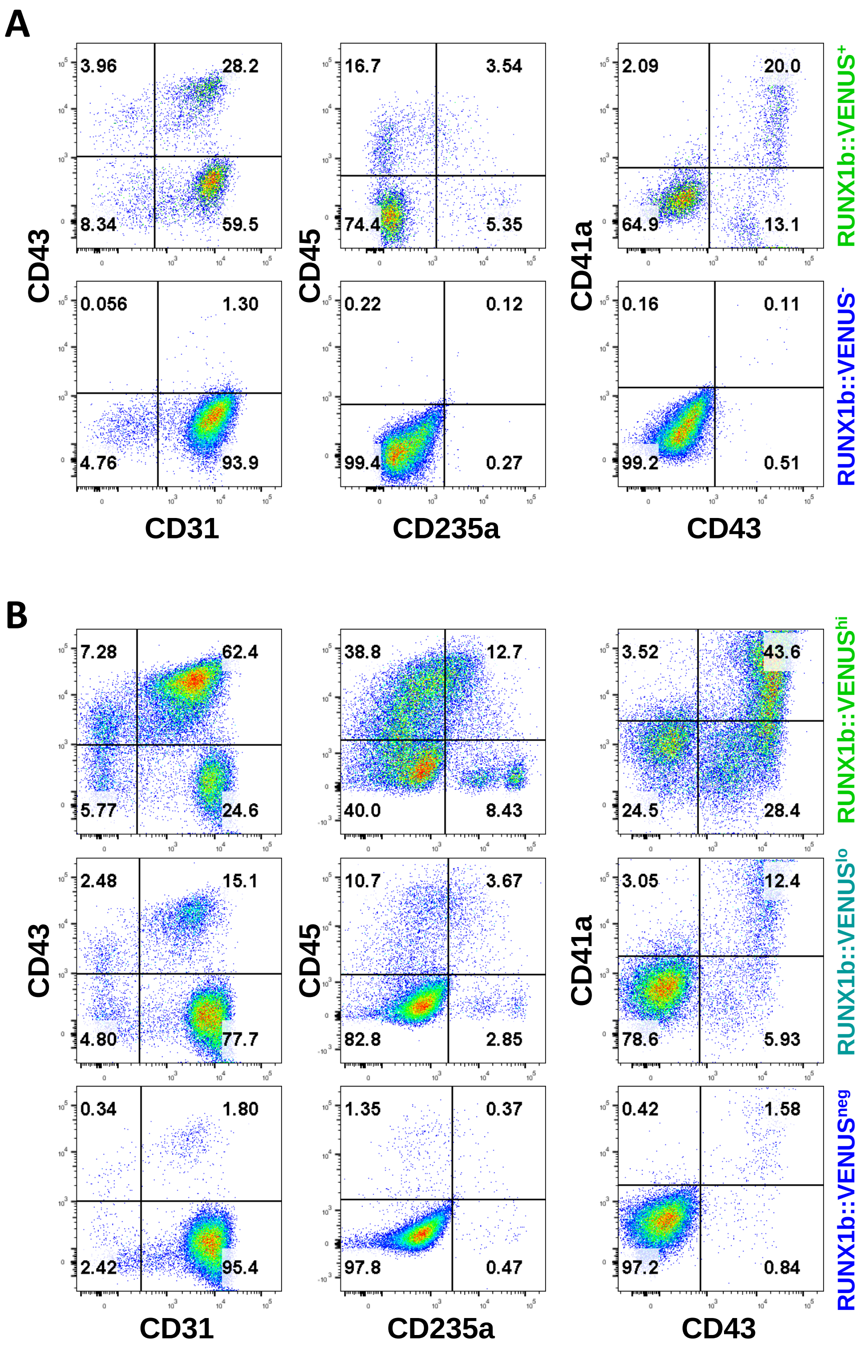

Supplementary Figure S2: **RUNX1b expression enriches for hemogenic potential.** (**A**) Flow cytometry analysis at day 7 of culture in hemogenic inducing condition for the sorted RUNX1b::VENUS negative and positive cell populations for the indicated cell surface markers. (**B**) Flow cytometry analysis at day 7 of culture in hemogenic inducing condition for the sorted RUNX1b::VENUS negative, low and high cell populations for the indicated cell surface markers.

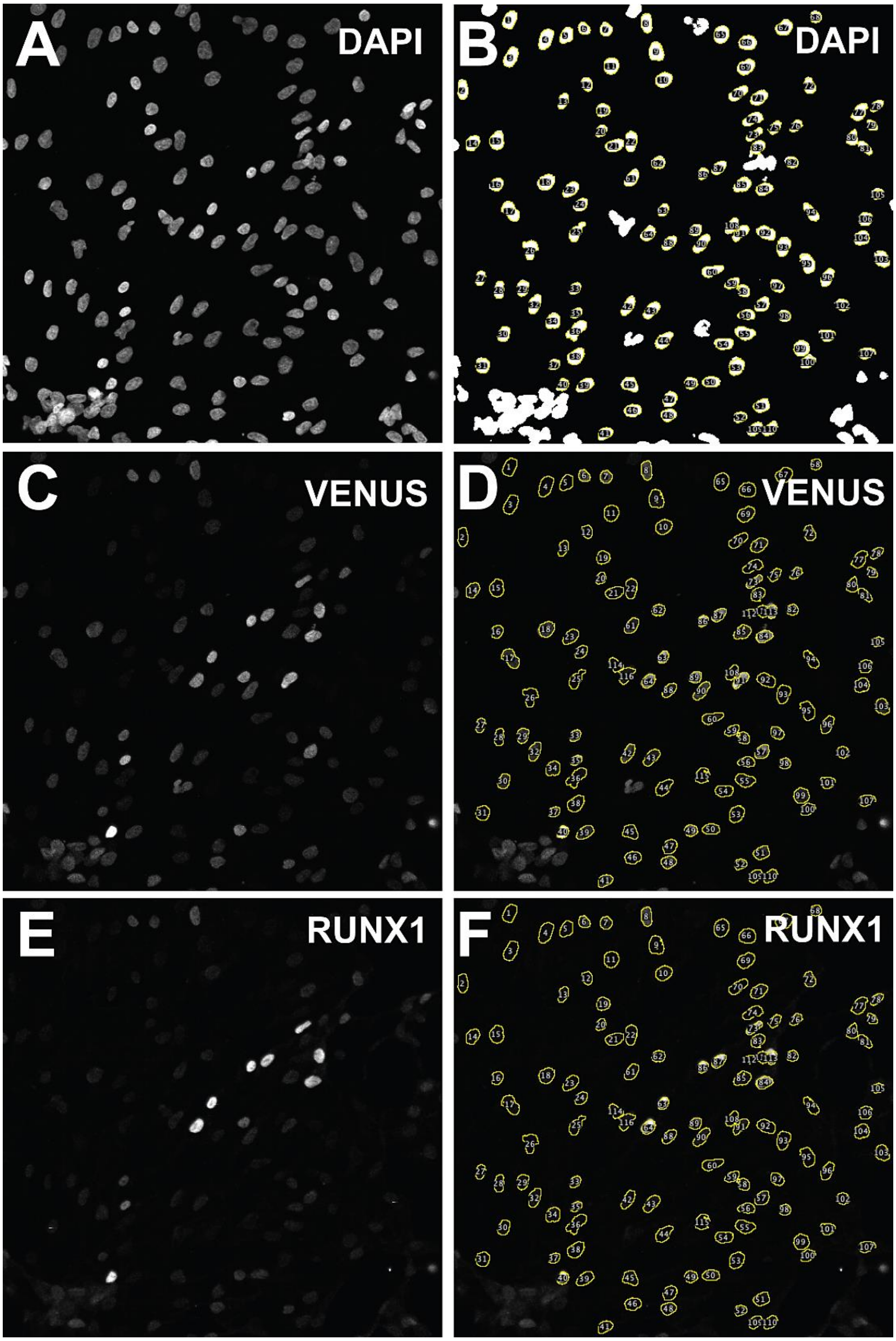

Supplementary Figure S3: **Integration of RUNX1b and VENUS signal in individual cells.** To quantify nuclear associated signal, DAPI images were used to detect nuclear regions of interest (ROIs). A threshold 8-bit binary image was created and 100+ isolated nuclei were detected by using the tracing tool in ImageJ to produce a collection of spatially calibrated ROIs (**A, B**). All ROIs were over-imposed to the VENUS (**C, D**) and RUNX1 (**E, F**). Integrated signal density was calculated for each ROI using the Measurement tool and plotted in GraphPad Prism.

Group	Lower	Estimate	Upper
$VENUS^{neg}$	1182	582.9	287.6
$VENUS^{neg}$	1792	806.7	363.1
$VENUS^{lo}$	162.4	112.2	77.53
$VENUS^{lo}$	126.7	87.6	60.53
$VENUS^{hi}$	25.3	17.2	11.72
$VENUS^{hi}$	18.6	12.5	8.46

Supplementary Figure S4 RUNX1b::VENUS expression enriches for hemogenic endothelium. Limiting dilution analysis of $CD31^+CD144^+CD43^-$ RUNX1b::VENUS negative, low and high populations isolated from EB day 6. Confidence intervals for HE frequency calculated using the ELDA online tool.

Supplemental text: materials and Methods

Generation of RUNX1 reporter hESC line: The hESCs were first transduced for RUNX1b-venus modification, neo resistant clones selected then the neo gene was removed. Next the Runx1b-venus hESCs were transduced for RUNX1c-Tomato, neo resistant selected and the Neo gene removed. Plasmid pSpCas9n(BB)-2A-GFP (PX461) was obtained from Addgene. Pairs of sgRNAs were designed using CHOPCHOP, WGE and E-CRISP to target intron 1 or 3 of the RUNX1 locus. Three pairs of sgRNAs were selected (see figure S1 for sgRNA sequences used) and purchased from Sigma to be ligated and cloned into PX461. To test the targeting efficiency of selected sgRNA pairs, HEK cells were transfected with the 3 different sgRNA-Cas9n plasmid pairs. Cells were plated in 10cm dishes at a density of $4x10^6$ cells/plate. A total volume of DNA of 4ml (2ul of sgRNA1-Cas9n + 2ul of sgRNA2-Cas9n) was added to 15ul polyethylenimine (PEI, Sigma) in 1ml DMEM and incubated at room temperature for 15-20 minutes. The transfection mix was then added to cell cultures for a 24-hour incubation period. After 3 days, GFP^+ cells were selected via FACS sort and genomic DNA was extracted for genotyping. In hESCs, CRISPR Cas9 technology was coupled with Homologous-Directed Repair strategy. Homology arms were amplified from human ESC-genomic DNA using Phusion High-Fidelity DNA Polymerase (NEB) or LongRange PCR kit (Qiagen) and cloned into pCR2.1-TOPO TA vector using TOPO TA cloning kit (Thermo Fisher Scientific). Homology arms were ligated into a donor plasmid containing either the H2B-Venus or Tomato reporter gene, preceded by IVS sequence, PGK-Neo cassette flanked by FRT sites, ampicillin resistance gene and pUC origin of replication. Man5 hESC cells (passage 58) were nucleofected with 1μg each of the selected sgRNA-Cas9n plasmid pair and 2μg of donor vector in Primary Solution P3 (Lonza), using program DN100 on Amaxa 4D Nucleofector (Lonza). After 2 days, GFP^+ cells were selected by FACS sort and plated on inactivated mouse embryonic fibroblast (MEFs) as single cells in 96-well plates, at a density of 2,200 cells/well in 12-well plates and 10^4 cells/dish in 10cm dishes. Clones were selected with hESC medium supplemented with 25μg/ml G418 (Thermo Fisher Scientific), expanded and genotyped. Heterozygote recombinant G418-resistant clones were nucleofected as described above with pCAG-Flpe:GFP plasmid (Addgene, #13788) to remove the PGK-Neo cassette. After 2 days, GFP^+ cells were sorted and plated on MEFs. Individual ESC clones were tested for heterozygous insertion of the reporter constructs by PCR for both endogenous and modify locus. Additionally, individual ESC clones were tested to determine if the two reporter constructs were inserted in cis or in trans on the RUNX1 locus, this was performed by RT-PCR to detect the transcription of both RUNX1c and RUNX1b isoforms on mRNA extracted from differentiated ESCs.

Immunofluorescence analysis: Sorted endothelial cells were grown on gelatin-coated glass slides (IBIDI) for 1 day in hemogenic inducing conditions then fixed with 4% formaldehyde, blocked and permeabilized with 5% goat serum and 0.3% Triton-X100. RUNX antibody (ab92336, Abcam) was used at 1:1,000, anti-rabbit ALEXA555 (A27039, Invitrogen) at 1:1,000 and Prolong Diamond antifade with DAPI (P36062, Invitrogen) as a mounting medium. Imaging acquisition was performed on a Leica SP8 inverted confocal microscope system using an HC PL APO CS2 40X/1.30 oil lens. 25 confocal planes (Z) were sequentially acquired for DAPI, VENUS and ALEXA555 emissions at 512x512, 16-bit pixels resolution and saved as .lif files. Imaging processing was performed in ImageJ (2.1.0/1.53c). Leica .lif file was opened using the bio-formats importer plugin, splitting the channels. Each channel was then Z-group projected at maximum intensity and pseudo-colored using the Hi-Lo Look Up Table (LUT) to avoid saturated pixels. To quantify nuclear associated signal, the DAPI image was used to detect nuclear regions of interest (ROIs). A threshold 8-bit binary image was created and 100+ isolated nuclei were detected by using the tracing tool to produce a collection of spatially calibrated ROIs. All ROIs were over-imposed to the VENUS and RUNX1 Z-group projected images in grey LUT. Integrated signal density (IntDen, arbitrary units) was calculated for each ROI using the Measurement tool and plotted in GraphPad Prism. VENUS/RUNX1 signal correlation was determined by the Pearson product-moment correlation coefficient (Pearson's coefficient) that ranged from 1 (total positive correlation) to −1 (total negative correlation) using GraphPad Prism analytical tools ($p<0.0001$, r 0.7570, R squared 0.5730).

Limiting dilution assays: LDA were performed in 96-well plates, which were previously coated with gelatin. An automatic cell deposition unit (Influx or Aria II/III/Fusion) was used to plate cells following a 1:2 serial dilution, covering a range from 500 to 4 cells. Cells were sorted in StemSpan supplemented with 5ng/ml VEGF, 5ng bFGF, 25ng/ml IGF1, 25ng/ml IGF2, 50ng/ml SCF, 50ng/ml TPO, 5ng/ml IL-11, 20ng/ml Flt3-L. After 7-14 days of culture, each well was scored for the presence of hematopoietic colonies and frequencies were calculated using the limdil function of the statistical package R.

RNA extraction and qPCR: Cell pellets were resuspended in 350ul of RLT plus buffer (Qiagen) supplemented with 5mM 2-mercaptoethanol and stored at -80°C. RNA samples were extracted with RNeasy Plus Mini Kit (Qiagen) and RNA concentration was measured with Nanodrop (Thermo Fisher Scientific). Synthesis of cDNA was performed using GoScript Reverse Transcription System (Promega) starting from 40-200ng of RNA sample. The product was diluted 1:5 and 5ul were taken to use in qPCR mix containing 10ul TaqMan Universal PCR Master Mix II (Applied Biosystems), 1ul of TaqMan Assay (see list below) and 4ul of dH_20 to a final volume of 20ul. Each sample was tested in triplicate. Reactions were run on StepOnePlus Real-Time PCR System (Life Technologies).

List of TaqMan Assays used for qPCR.

TaqMan Assay #	Supplier	Species
**Hs02558380_s1 RUNX1B/C**	Thermo Fisher Scientific	Human
**Hs01021967_m1 RUNX1C**	Thermo Fisher Scientific	Human
**Hs04186042_m1 RUNX1A**	Thermo Fisher Scientific	Human
**Hs00187842_m1 B2M**	Thermo Fisher Scientific	Human
